# Monocyte Chemotactic Proteins (MCP) in Colorectal Adenomas Are Differently Expressed at the Transcriptional and Protein Levels: Implications for Colorectal Cancer Prevention

**DOI:** 10.3390/jcm10235559

**Published:** 2021-11-26

**Authors:** Jarosław Wierzbicki, Artur Lipiński, Iwona Bednarz-Misa, Łukasz Lewandowski, Katarzyna Neubauer, Paulina Lewandowska, Małgorzata Krzystek-Korpacka

**Affiliations:** 1 Department of Minimally Invasive Surgery and Proctology, Wroclaw Medical University, 50-556 Wroclaw, Poland; 2 Department of Clinical Pathology, Wroclaw Medical University, 50-556 Wroclaw, Poland; artur.lipinski@umw.edu.pl; 3 Department of Medical Biochemistry, Wroclaw Medical University, 50-368 Wroclaw, Poland; iwona.bednarz-misa@umw.edu.pl (I.B.-M.); lukasz.lewandowski@umw.edu.pl (Ł.L.); p.lewandowska2803@gmail.com (P.L.); 4 Department of Gastroenterology and Hepatology, Wroclaw Medical University, 50-556 Wroclaw, Poland; katarzyna.neubauer@umw.edu.pl

**Keywords:** colorectal cancer, chemoprevention, macrophage-associated chemokines, CCL2, CCL7, CCL8, colorectal adenoma

## Abstract

The expression of monocyte chemotactic proteins (MCPs) in colorectal polyps and their suitability as targets for chemoprevention is unknown, although MCP expression and secretion can be modulated by non-steroidal inflammatory drugs. This study was designed to determine the expression patterns of MCP-1/*CCL2*, MCP-2/*CCL8*, and MCP-3/*CCL7* at the protein (immunohistochemistry; *n* = 62) and transcriptional levels (RTqPCR; *n* = 173) in colorectal polyps with reference to the polyp malignancy potential. All chemokines were significantly upregulated in polyps at the protein level but downregulated at the transcriptional level by 1.4-(*CCL2*), 1.7-(*CCL7*), and 2.3-fold (*CCL8*). There was an inverse relation between the immunoreactivity toward chemokine proteins and the number of corresponding transcripts in polyps (*CCL2* and *CCL7*) or in normal mucosa (*CCL8*). The downregulation of chemokine transcripts correlated with the presence of multiple polyps (*CCL2* and *CCL8*), a larger polyp size (*CCL2*, *CCL7*, and *CCL8*), predominant villous growth patterns (*CCL2*, *CCL7* and *CCL8*), and high-grade dysplasia (*CCL2* and *CCL8*). In conclusion, MCP-1/*CCL2*, MCP-2/*CCL8*, and MCP-3/*CCL7* chemokines are counter-regulated at the protein and transcriptional levels. Chemokine-directed chemopreventive strategies should therefore directly neutralize MCP proteins or target molecular pathways contributing to their enhanced translation or reduced degradation, rather than aiming at *CCL2*, *CCL7* or *CCL8* expression.

## 1. Introduction

Colorectal cancer (CRC) is one of the most common malignancies worldwide, the incidence of which has recently been reduced, owing to improved screening. Chemoprevention, which is using chemicals to prevent, delay, or reverse the carcinogenesis, is another intensively studied strategy for reducing CRC risk in high-risk people as well as the general population [1]. Patients with inflammatory bowel disease (IBD), encompassing Crohn’s disease and ulcerative colitis, are more likely to develop cancer, and the risk increases with increasing disease duration, extension, and severity, as well as the occurrence of primary sclerosing cholangitis or inflammatory pseudopolyps [2]. Most sporadic CRCs, however, develop from polyps via the classic adenoma-carcinoma sequence or, less commonly, the serrated polyp pathway [3]. This well-known and relatively slow progression makes CRC particularly open to chemoprevention. Therefore, a better understanding of the molecular profile of polyps as premalignant lesions and recognition of the features distinguishing those with high potential for malignancy is considered a prerequisite for developing effective surveillance and chemopreventive strategies [3].

Inflammation plays a well-established role in cancer development as a facilitator of tumor cell proliferation, survival, and migration and an enabler of cancer angiogenesis and metastasis [4]. While chronic inflammation is also a main trigger of neoplastic transformation in IBD patients [2], the contribution of inflammatory mediators to adenoma-carcinoma transition is less evident and poorly understood. Nonetheless, it has been increasingly recognized [5]. Pre-cancerous lesions have been shown to be infiltrated by immune cells in proportion to the dysplasia grade and adenoma size [6], implicating the involvement of chemokines. Consistently, chemokine decoy receptors—ACKR2 and 4—have been downregulated in colorectal adenomas, and their expression was inversely related with the malignancy potential [7]. Recent studies have shown a link between systemic inflammatory mediators, including macrophage-associated chemokines [8] and colorectal adenomas [9,10]. Moreover, non-steroidal anti-inflammatory drugs (NSAIDs) have been repeatedly shown to reduce the incidence and size of colorectal neoplasms [1,11,12,13,14] via cyclooxygenase/prostaglandin E2 pathway-dependent and independent mechanisms [15], further stressing the relevance of inflammation in colorectal carcinogenesis.

Macrophage-associated chemokines are gaining attention as potential targets for anti-neoplastic therapies [16]. Monocyte and macrophage chemotactic proteins (MCPs) belong to the C-C subfamily of chemokines and are expressed by epithelial, endothelial, stromal, and immune cells. They display activities that may support tumor growth both indirectly and directly, acting either in an autocrine or paracrine manner [17,18,19,20]. The expression of *CCL2*/MCP-1, an MCP protagonist, has been shown to increase along with an advancing CRC stage [21,22], and a close link between the chemokine and the invasion and metastasis [23] has been confirmed in functional studies [24]. However, depending on the chemokine, context, and type of recruited cells, MCP chemokines may also strengthen anti-tumor responses [17,18,19,20]. Accordingly, transfection with *CCL7*/MCP-3 hampered tumor growth and prevented metastasis in animal models of cancer [25,26].

The MCP chemokine status in colorectal adenomas is largely unknown but indicative of counter-regulation during normal mucosa-adenoma and adenoma-carcinoma transitions [27]. Our group has recently demonstrated that the expression of MCP chemokines can be modulated by novel NSAIDs belonging to the oxicam class (manuscript submitted). Therefore, this study was conducted to assess the suitability of MCP chemokines as targets for chemoprevention by determining the expression patterns of MCP-1/*CCL2*, MCP-2/*CCL8*, and MCP-3/*CCL7* in colorectal polyps and the chemokine association with polyp potential for malignancy.

## 2. Materials and Methods

### 2.1. Immunohistochemistry (IHC)

#### 2.1.1. Patients

Tissue samples of the large intestine lesions were routinely collected during endoscopic examination from 66 patients admitted into the Department of Minimally Invasive Surgery and Proctology of Wroclaw Medical University and evaluated by the Department of Clinical Pathology of Wroclaw Medical University. Following histopathological examination, based on hematoxylin-eosin staining (HE), two patients were diagnosed with celiac disease, one with adenocarcinoma, and one with inflammatory polyps, and they were therefore excluded from further examination. Detailed characteristics of the remaining patients are given in Table 1 (IHC cohort).

Patient-matched samples of macroscopically normal mucosa were collected 10–15 cm from the lesions and served as controls. Sample collection was approved by the medical ethics committees of Wroclaw Medical University (#KB-247/2018 from 24 April 2018).

#### 2.1.2. Sample Handling

Th collected tissue samples were preserved in 4% buffered formalin solution for 24–72 h and subsequently subjected to a dewatering process in a Tissue-Tek Xpress^®^ x120 microwave tissue processor (Sakura Finetek, Alphen aan den Rijn, The Netherlands) using the Xpress Reagent set (Sakura Finetek; cat. No 7730). After being embedded in paraffin to create blocks, the tissue samples were sliced into 3-μm thick fragments.

#### 2.1.3. IHC Procedure

The 3-μm thick tissue fragments were placed on a microscope slide with an additional adhesive surface. Cusabio Technology LLC (Houston, TX, USA) rabbit monoclonal anti-human antibodies directed against the corresponding chemokine were used for the immunohistochemical reactions, consisting of *CCL2* Antibody (cat. No CSB-PA05865A0Rb), *CCL7* Antibody (cat. No CSB-PA082817), and *CCL8* Antibody (cat. No CSB-PA07629A0Rb).

The first stage was the dewaxing of tissue fragments using the PT Link system (Agilent Technologies Inc., Carpinteria, CA, USA) by boiling in a high-pH buffer at 97 °C. The next stage (i.e., antibody application) took place automatically using the Autostainer Plus Link platform (Agilent Technologies Inc.) according to the protocol provided by the antibodies’ manufacturer. The effects of all reactions were visualized with the EnVision FLEX set (Agilent Technologies Inc.; cat. No K800221-2) using a 3, 39-diaminobenzidine tetrahydrochloride (DAB) chromogen, which turned brown in the case of a positive reaction. Hematoxylin was used for counterstaining.

Photos were taken under an Olympus System BX51 microscope with a U0TV.63XC digital camera (Olympus Corporation, Tokyo, Japan).

#### 2.1.4. IHC Scoring

In accordance with the characteristics of the antibodies used, a granular cytoplasmic reaction occurred in all cases. The basis for the assessment was a reaction in the cytoplasm of glandular epithelial cells, which forms the mucous membrane of the large intestine, as well as adenomas, regardless of their histological type.

The reaction results were classified as follows:

- Score 0, a negative result: either no reaction occurred in the glandular epithelial cells, or it only occurred in the stromal area of the lesion or control tissue fragment;
- Score 1, a weak positive result (+): such cases exhibited a weak cytoplasmic reaction (a low-intensity one), or the reaction did not encompass the entire lesion or control tissue fragment;
- Score 2, a positive result (++): a strong cytoplasmic reaction encompassing the entire lesion or the entire control tissue epithelium.

### 2.2. Transcriptional Analysis (Reverse-Transcribed Quantitative Polymerase Chain Reaction (RTqPCR))

#### 2.2.1. Patients

Paired samples of polyp and normal mucosa from 176 patients admitted to the Department of Minimally Invasive Surgery and Proctology or to the Department of Gastroenterology and Hepatology of Wroclaw Medical University were included in the current study (Medical Ethics Committees of Wroclaw Medical University approval #KB-247/2018 from 24 April 2018). Of those, 66 patients were common with the IHC cohort, and 3 of them were excluded from further analysis for the reasons stated previously (2 cases of celiac disease and 1 inflammatory polyp). The characteristics of the RTqPCR cohort are presented in Table 1.

#### 2.2.2. Sample Handling

The tissue samples were rinsed with PBS, soaked in RNAlater (Ambion Inc., Austin TX, USA), and kept frozen at −80 °C until RNA extraction. 

#### 2.2.3. RNA Isolation, cDNA Synthesis, and Quantitative PCR (qPCR)

After thawing, the samples (up to 40 mg) were homogenized in Fastprep 24 Homogenizer (MP Biomedical, OH, USA) using ceramic spheres and a lysis buffer (PureLink™ RNA Mini Kit from Invitrogen, Thermo Fisher Scientific, Waltham, MA, USA) supplemented with β-mercaptoethanol (Sigma Aldrich, St. Luis, MO, USA) at 1:10 (*v*/*v*).

The total RNA was isolated using phenol-chloroform extraction and purified with a PureLink™ RNA Mini Kit (Invitrogen). On-column digestion with DNase (PureLink™ DNase Set (Invitrogen) was applied to avoid contamination with the genomic DNA. The RNA was quantified in the extract, and its purity as well as integrity were assessed with a NanoDrop 2000 spectrophotometer (Thermo-Fisher Scientific, Waltham, MA, USA) and LabChip microfluidic technology using the Experion platform and Experion RNA StdSens analysis kits (BioRad, Herkules CA, USA).

An iScript™ cDNA Synthesis Kit (BioRad, Herkules CA, USA) and C1000 thermocycler (BioRad) were used to reverse transcribe the obtained RNA (1000 ng per reaction).

The relative number of transcripts was determined by quantitative polymerase chain reactions (qPCRs) using the CFX96 Real-Time PCR system (BioRad, Herkules CA, USA) under the following cycling conditions: 30 s activation at 95 °C, 5 s denaturation at 95 °C, and annealing and extension for 5 s at 61 °C for 40 cycles. Product specificity was ensured by melting curve analysis (60–95 °C with fluorescent readings every 0.5 °C) and an electrophoresis in agarose with SYBR Green detection. The reaction mixture consisted of 10 nM forward and reverse target-specific primers (1 µL of each; Genomed, Warsaw, Poland), 2 × SsoFast EvaGreen^®^ Supermix (10 µL; BioRad, Herkules CA, USA), a cDNA template (2 µL, diluted 1:5), and water up to 20 µL. The intron-spanning starter sequences were as follows: 5′-tctgtgcctgctgctcatag-3′ and 5′-acttgctgctggtgattcttc-3′ (*CCL2* forward and reverse; product size: 155 bp); 5′-acagaaggaccaccagtagcca-3′ and 5′-ggtgcttcataaagtcctggacc-3′ (*CCL7* forward and reverse; product size: 117 bp); 5′-tatccagaggctggagagctac-3′ and 5′-tggaatccctgacccatctctc-3′ (*CCL8* forward and reverse; product size: 128 bp); 5′-ggcaaatgctggacccaacaca-3′ and 5′-tgctggtcttgccattcctgga-3′ (*PPIA* forward and reverse; product size: 161 bp); 5′-tcacaacaagcataccaagaagc-3′ and 5′-gtatccgatgtccacaatgtcaag-3′ (*RPLP0* forward and reverse; product size: 263 bp); and 5′-tagattattctctgatttggtcgtattgg-3′ and 5′-gctcctggaagatggtgatgg-3′ (*GAPDH* forward and reverse; product size: 223 bp). Except for *CCL2* and *GAPDH*, being designed using Beacon Designer Probe/Primer Design Software (BioRad) and validated in silico (Blast analysis), the starters’ sequences were proposed by OriGene (www.origene.com (accessed on 1 March 2021)).

The Cq values of the technical replicates were averaged prior to expression analysis. For each analyzed gene, the geometric mean of all Cq values was subtracted from the sample Cq, yielding ΔCq. The ΔCq values were then linearized by 2^ΔCq^ conversion and normalized to the internal control: the geometric mean of *PPIA* and *RPLP0* expression [28] or *GAPDH* expression. The resulting normalized relative quantity values were denoted NRQ [29] and subjected to statistical analysis.

### 2.3. Data Analysis

All statistical analyses were conducted using MedCalc^®^ Statistical Software version 20.011 (MedCalc Software Ltd., Ostend, Belgium). The level of statistical significance was set at ≤0.05, and all calculated *p* values were two-sided.

The distribution of data and homogeneity of variances were tested prior to the analyses using the Kolmogorov–Smirnov test and Levene test, respectively. The expression data, following log transformation, were analyzed using a *t*-test for the paired samples and a *t*-test for the independent samples with Welch correction in case of unequal variances or one-way ANOVA (multigroup comparisons) with Student–Newman–Keuls post hoc analysis. The IHC data were analyzed with the Wilcoxon test. Frequency analysis was conducted with a chi-squared test and correlation analysis with Pearson moment (*r*) or Spearman rank (*ρ*) correlation. The contingency coefficient (*C*) was calculated to express association for the categorical data. The stepwise method of linear multivariate regression was applied to identify the independent predictors of the dependent variable, with variables entered into the regression model if *p* < 0.05 and removed if *p* > 0.1.

## 3. Results

### 3.1. Expression of MCP Chemokines at the Protein Level

#### 3.1.1. MCP Proteins in Colorectal Adenomas

**Figure 1:**
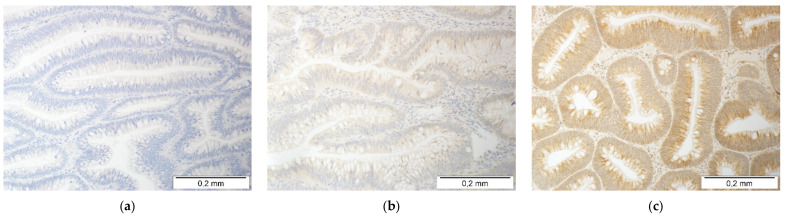
Immunohistochemical analysis of MCP-1/*CCL2* protein expression in colorectal adenomas: (**a**) negative reaction, with no reaction in the glandular epithelial cells or it only occurring in the stromal area of the lesion; (**b**) a weak cytoplasmic reaction, or the reaction did not encompass the entire lesion (score 1; +); and (**c**) a strong cytoplasmic reaction encompassing the entire lesion (score 2; ++). The tissue slides were incubated with rabbit anti-human MCP-1/*CCL2* antibodies with a DAB chromogen, stained brown in the case of a positive reaction and counterstained with hematoxylin. All the photos were taken at 200×.

Protein expression between the normal and polyp tissue was compared in 62 patients. Regarding MCP-1/*CCL2*, 8.1% of patients had lower protein expression in the polyps than the corresponding normal tissue, 40.3% had comparable expression levels, and 51.6% had higher MCP-1/*CCL2* expression in the polyps than the matching normal tissue. Pairwise comparison showed significant MCP-1/*CCL2* overexpression in the polyps (*p* = 0.0002). Photos representing various immunoreactivities of the polyps toward MCP-1/*CCL2* are presented in Figure 1.

**Figure 2:**
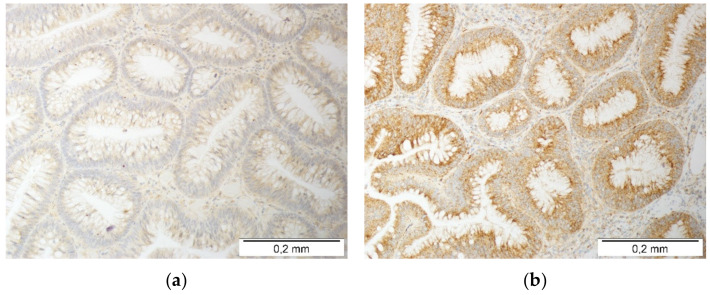
Immunohistochemical analysis of MCP-3/*CCL7* protein expression in colorectal adenomas: (**a**) a weak cytoplasmic reaction, or the reaction did not encompass the entire lesion (score 1; +) and (**b**) a strong cytoplasmic reaction encompassing the entire lesion (score 2; ++). There were no polyp samples negative for MCP-3/*CCL7* in the tested cohort. The tissue slides were incubated with rabbit anti-human MCP-3/*CCL7* antibodies with a DAB chromogen, stained brown in the case of a positive reaction and counterstained with hematoxylin. All the photos were taken at 200×.

Regarding MCP-3/*CCL7*, 8.1% of patients had lower protein expression in the polyps than the corresponding normal tissue, 58.1% had comparable expression levels, and 33.9% had higher MCP-3/*CCL7* expression in the polyps than the matching normal tissue. Still, pairwise comparison showed significant MCP-3/*CCL7* overexpression in the polyps (*p* = 0.003). There were no polyp samples negative for MCP-3/*CCL7* in the tested cohort. Photos representing immunoreactivity scores of one and two toward MCP-3/*CCL7* are presented in Figure 2.

**Figure 3:**
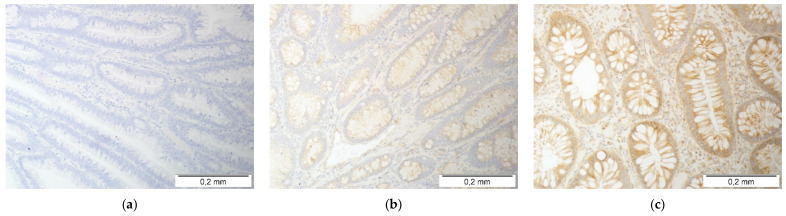
Immunohistochemical analysis of MCP-2/*CCL8* protein expression in colorectal adenomas: (**a**) negative reaction, showing either no reaction in the glandular epithelial cells or it only occurring in the stromal area of the lesion; (**b**) a weak cytoplasmic reaction, or the reaction did not encompass the entire lesion (score 1; +); and (**c**) a strong cytoplasmic reaction encompassing the entire lesion (score 2; ++). The tissue slides were incubated with rabbit anti-human MCP-2/*CCL8* antibodies with a DAB chromogen, stained brown in the case of a positive reaction and counterstained with hematoxylin. All the photos were taken at 200×.

Regarding MCP-2/*CCL8*, 6.5% of patients had lower protein expression in the polyps than the corresponding normal tissue, 40.3% had comparable expression levels, and 53.2% had higher MCP-2/*CCL8* expression in the polyps than the matching normal tissue. The pairwise comparison showed significant MCP-2/*CCL8* overexpression in the polyps (*p* < 0.0001). Photos representing various immunoreactivities of the polyps toward MCP-2/*CCL8* are presented in Figure 3.

#### 3.1.2. MCP Association with the Anatomical Site and Pathological Findings

MCP-1/*CCL2*, MCP-3/*CCL7*, and MCP-2/*CCL8* protein expression in the polyps was not associated with the polyp histology or grade of dysplasia. The exception was MCP-1/*CCL2*, for which low immunoreactivity was significantly associated with low-grade dysplasia and higher immunoreactivity with high-grade dysplasia (Table 2). The polyp size and its anatomical location had no effect on MCP-1/*CCL2*, MCP-3/*CCL7*, or MCP-2/*CCL8* protein expression in the neoplasm (Table 2).

The change in expression between the normal and neoplastic mucosa (decreased, unaffected, or increased) with reference to the histological type, dysplasia grade, size, and anatomical location did not show significant associations, except for MCP-3/*CCL7* and the anatomical site. Higher MCP-3/*CCL7* immunoreactivity in the polyps than the adjacent mucosa was more likely to accompany left-sided polyps, and no change in immunoreactivity was more likely for the right-sided polyps (contingency coefficient (*C*) = 0.372, *p* = 0.041).

The immunoreactivity for MCP-1/*CCL2* in the polyps positively correlated with that for MCP-2/*CCL8* (*ρ* = 0.46, *p* = 0.0001), while MCP-1/*CCL2′*s correlation with MCP-3/*CCL7* was weak and borderline significant (*ρ* = 0.25, *p* = 0.047), and that between MCP-3/*CCL7* and MCP-2/*CCL8* was non-significant (*ρ* = 0.25, *p* = 0.053).

### 3.2. Expression of MCP Chemokines at the Transcriptional Level

#### 3.2.1. MCP Transcripts in Colorectal Adenomas

*CCL2*, *CCL7*, and *CCL8* mRNA expression was analyzed against *GAPDH* or the geometric mean of a pair of normalizers: *PPIA* and *RPLP0*. Regardless the normalization strategy, the average *CCL2*, *CCL7*, and *CCL8* expression was lower in the polyps than the normal mucosa and significantly lower in the case of *CCL7* and *CCL8* (Table 3).

As these results contradicted those obtained for the MCP proteins, an additional 110 patient-matched pairs of polyp and normal mucosa samples were analyzed by RTqPCR. Like for the IHC cohort alone, *CCL2*, *CCL7*, and *CCL8* were downregulated in the polyps compared with the normal mucosa, and the degree of downregulation was similar between the smaller and larger cohorts and between the normalizers (Table 3).

Further analyses were conducted on the enlarged cohort (*n* = 173) using *PPIA*/*RPLP0* as normalizers.

#### 3.2.2. Effect of the Polyp Size on the Expression of MCP Chemokines (mRNA)

**Figure 4:**
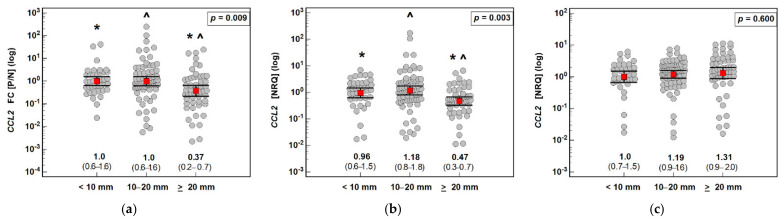

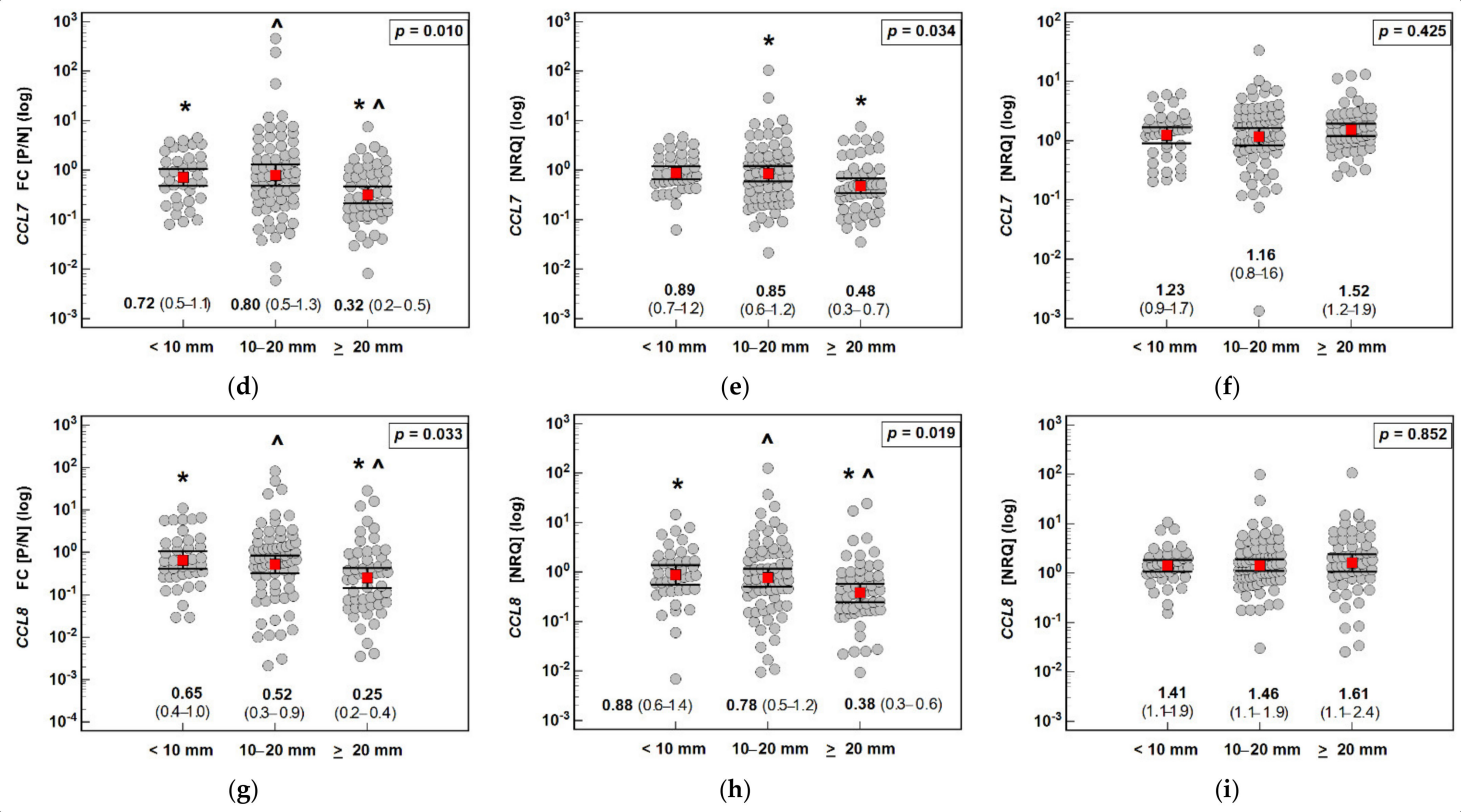
Effect of polyp size on the expression (mRNA) of MCP chemokines in patients with colorectal polyps: (**a**) fold change (FC) in *CCL2* expression between the polyp and normal mucosa (P/N); (**b**) *CCL2* expression in a polyp; (**c**) *CCL2* expression in normal tissue; (**d**) fold change (FC) in *CCL7* expression between the polyp and normal mucosa (P/N); (**e**) *CCL7* expression in a polyp; (**f**) *CCL7* expression in normal tissue; (**g**) fold change (FC) in *CCL8* expression between the polyp and normal mucosa (P/N); (**h**) *CCL8* expression in a polyp; (**i**) *CCL8* expression in normal tissue. Data analyzed as logarithms using one-way ANOVA with a Student–Newman–Keuls post hoc test and presented as means with 95% confidence intervals (red squares with whiskers and numbers below the dot plots). Significant (*p* < 0.05) differences between groups (identified in post hoc analysis) are indicated by the same symbol type (*, ^). NRQ: normalized relative quantity.

The fold change in *CCL2*, *CCL7*, and *CCL8* expression was significantly affected by the polyp size. *CCL2* expression was downregulated solely in the largest polyps (by 2.7-fold). In the case of *CCL7* (3.1-fold) and *CCL8* (4-fold), the downregulation was more marked in the larger polyps than the smaller polyps. Detailed analysis showed that gene expression was non-significantly higher in the normal mucosa of the patients with the largest polyps while being significantly lower in their polyps (by 2.1-fold for *CCL2* and *CCL7* and 2.7-fold for *CCL8*) (Figure 4).

#### 3.2.3. Effect of the Polyp Type on the Expression of MCP Chemokines (mRNA)

**Figure 5:**
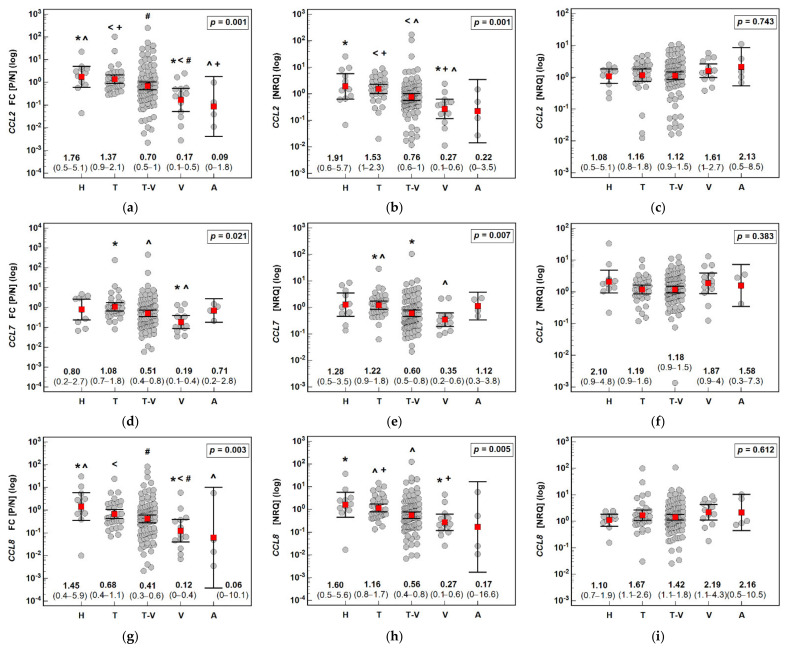
Effect of the polyp type on the expression (mRNA) of MCP chemokines in patients with colorectal polyps: (**a**) fold change (FC) in *CCL2* expression between the polyp and normal mucosa (P/N); (**b**) *CCL2* expression in a polyp; (**c**) *CCL2* expression in normal tissue; (**d**) fold change (FC) in *CCL7* expression between the polyp and normal mucosa (P/N); (**e**) *CCL7* expression in a polyp; (**f**) *CCL7* expression in normal tissue; (**g**) fold change (FC) in *CCL8* expression between the polyp and normal mucosa (P/N); (**h**) *CCL8* expression in a polyp; (**i**) *CCL8* expression in normal tissue. Data analyzed as logarithms using one-way ANOVA with a Student–Newman–Keuls post hoc test and presented as means with 95% confidence intervals (red squares with whiskers and numbers below dot plots). Significant (*p* < 0.05) differences between groups (identified in post hoc analysis) are indicated by the same symbol type (*, ^, <, +, #). NRQ: normalized relative quantity; H: hyperplastic polyps; T: tubular adenomas; T-V: tubulo-villous adenomas; V: villous adenomas; A: adenocarcinoma in a polyp.

The polyp type had a significant impact on the fold change in *CCL2*, *CCL7*, and *CCL8* expression. For *CCL2* and *CCL8*, it was the highest and indicative of gene upregulation (by 1.8- and 1.5-fold, respectively) in the polyp compared with the corresponding normal mucosa in hyperplastic polyps and the lowest in adenocarcinoma in the polyp, being indicative of gene downregulation in the polyp compared with the normal mucosa (by 11.1- and 16.7-fold, respectively). The fold change in *CCL2*, *CCL7*, and *CCL8* expression decreased gradually along with an increasing villous component in the adenomas. *CCL2* was upregulated in the tubular adenomas by 1.4-fold compared with the normal tissue but downregulated in the tubulo-villous and villous adenomas by 1.4- and 5.9-fold, respectively. *CCL7* expression in the tubular adenomas was comparable to normal tissue but downregulated in the tubulo-villous and villous adenomas by 2.0- and 5.3-fold, respectively. *CCL8* was already downregulated in the tubular adenomas (by 1.5-fold) but more markedly so in the tubulo-villous and villous adenomas, in which *CCL8* was downregulated by 2.4- and 8.3-fold, respectively (Figure 5).

Detailed analysis of the gene expression patterns in the normal mucosa and polyps showed that the *CCL2*, *CCL7*, and *CCL8* expression in the normal tissue was comparable and not affected by the polyp type, contrary to gene expression in the polyps. *CCL2*, *CCL7*, and *CCL8* expression was the highest in the hyperplastic polyps and lower in the adenomas, in which it gradually decreased along with an increasing villous component. The polyp expression of *CCL2* and *CCL8* (but not *CCL7*) was the lowest in the adenocarcinomas (Figure 5).

#### 3.2.4. Effect of the Dysplasia Grade on the Expression of MCP Chemokines (mRNA)

**Figure 6:**
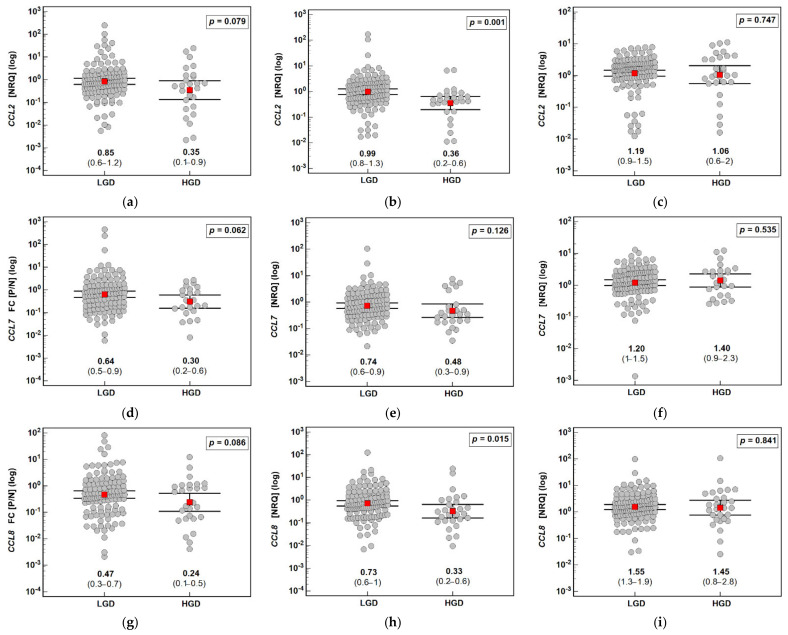
Effect of the dysplasia grade on the expression (mRNA) of MCP chemokines in patients with colorectal polyps: (**a**) fold change (FC) in *CCL2* expression between the polyp and normal mucosa (P/N); (**b**) *CCL2* expression in a polyp; (**c**) *CCL2* expression in normal tissue; (**d**) fold change (FC) in *CCL7* expression between the polyp and normal mucosa (P/N); (**e**) *CCL7* expression in a polyp; (**f**) *CCL7* expression in normal tissue; (**g**) fold change (FC) in *CCL8* expression between the polyp and normal mucosa (P/N); (**h**) *CCL8* expression in a polyp; (**i**) *CCL8* expression in normal tissue. Data analyzed as logarithms using a t-test for independent samples with Welch correction in the case of unequal variances and presented as means with 95% confidence intervals (red squares with whiskers and numbers below dot plots). NRQ: normalized relative quantity; LGD: low-grade dysplasia; HGD: high-grade dysplasia.

*CCL2*, *CCL7* and *CCL8* expression tended to decrease in the polyps compared with the normal tissue, though more markedly in the case of adenomas with high-grade dysplasia. *CCL2* expression in the adenomas with high-grade dysplasia was significantly decreased compared with the adenomas with low-grade dysplasia (by 2.8-fold). Likewise, *CCL8* expression was lower by 2.2-fold (Figure 6).

#### 3.2.5. Effect of the Number of Polyps on the Expression of MCP Chemokines (mRNA)

**Figure 7:**
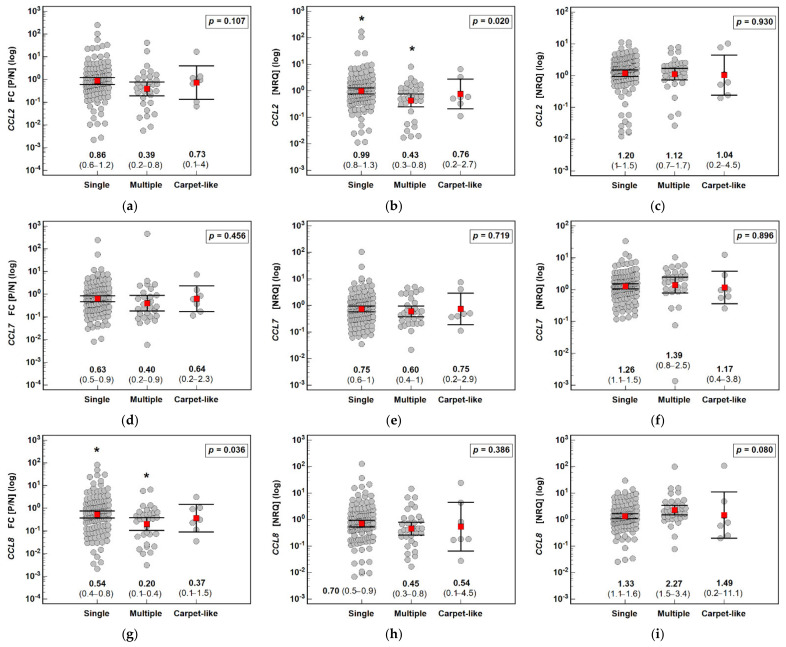
Effect of the number of polyps on the expression (mRNA) of MCP chemokines in patients with colorectal polyps: (**a**) fold change (FC) in *CCL2* expression between the polyp and normal mucosa (P/N); (**b**) *CCL2* expression in a polyp; (**c**) *CCL2* expression in normal tissue; (**d**) fold change (FC) in *CCL7* expression between the polyp and normal mucosa (P/N); (**e**) *CCL7* expression in a polyp; (**f**) *CCL7* expression in normal tissue; (**g**) fold change (FC) in *CCL8* expression between the polyp and normal mucosa (P/N); (**h**) *CCL8* expression in a polyp; (**i**) *CCL8* expression in normal tissue. Data analyzed as logarithms using one-way ANOVA with a Student–Newman–Keuls post hoc test and presented as means with 95% confidence intervals (red squares with whiskers and numbers below dot plots). Significant (*p* < 0.05) differences between groups (identified in post hoc analysis) are indicated by an asterisk (*). NRQ: normalized relative quantity.

Based on the number of polyps and their character (protruding or flat lesions), patients were classified as having single, multiple, or carpet-like polyps. Only polyp *CCL2* expression was significantly affected by the number of polyps; it was significantly lower in patients with multiple lesions than those with single polyps (by 2.3-fold). In turn, *CCL8* expression in normal mucosa tended to be higher in patients with multiple polyps, resulting in more pronounced downregulation (5-fold in the case of multiple lesions and 1.9-fold in the case of single polyps) (Figure 7).

#### 3.2.6. Association of Cumulative Risk of Adenoma-to-Adenocarcinoma Transformation with the Expression of MCP Chemokines (mRNA)

As the risk of adenoma transformation to adenocarcinoma is higher in the case of larger and multiple polyps and those with dominant villous growth patterns and high-grade dysplasia, we summarized those risk factors by calculating a cumulative risk factor. To ensure the same weight for all risk factors, three categorical variables such as tubular, tubulo-villous, and villous adenomas or <10 mm, 10–19 mm, and ≥20 mm adenomas were assigned scores of 1, 1.5, and 2, respectively, and two categorical variables such as low- and high-grade dysplasia or single and multiple adenomas were assigned scores of 1 and 2, respectively.

Both the fold change in expression of *CCL2*, *CCL7*, and *CCL8* and gene expression in adenoma were inversely correlated with a cumulative risk of transformation. The strongest association was noted for adenoma expression of *CCL2*. In the case of *CCL7*, its expression concomitantly decreased in adenoma and increased in macroscopically normal tissue (Table 4).

#### 3.2.7. Effect of the Anatomical Subsite on the Expression of MCP Chemokines (mRNA)

**Figure 8:**
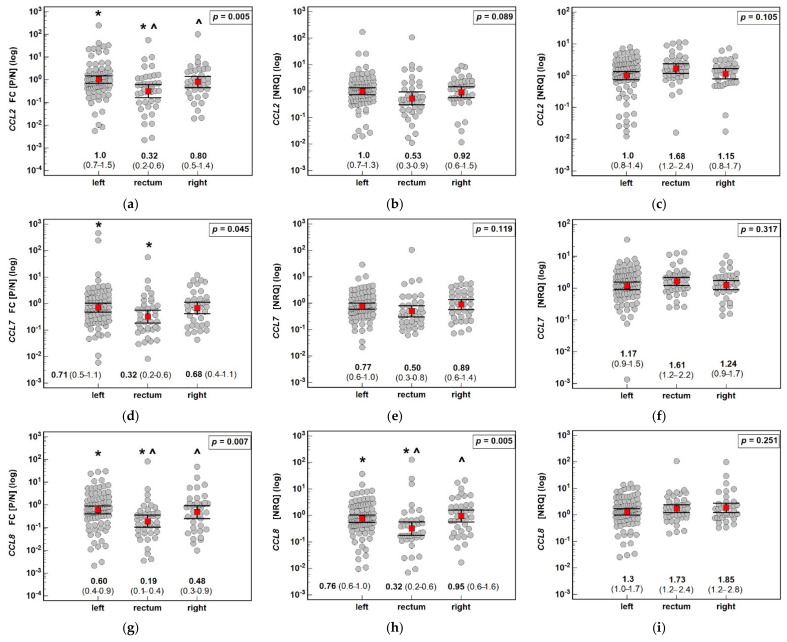
Effect of the anatomical subsite on the expression (mRNA) of MCP chemokines in patients with colorectal polyps: (**a**) fold change (FC) in *CCL2* expression between the polyp and normal mucosa (P/N); (**b**) *CCL2* expression in a polyp; (**c**) *CCL2* expression in normal tissue; (**d**) fold change (FC) in *CCL7* expression between the polyp and normal mucosa (P/N); (**e**) *CCL7* expression in a polyp; (**f**) *CCL7* expression in normal tissue; (**g**) fold change (FC) in *CCL8* expression between the polyp and normal mucosa (P/N); (**h**) *CCL8* expression in a polyp; (**i**) *CCL8* expression in normal tissue. Data analyzed as logarithms using one-way ANOVA with a Student–Newman–Keuls post hoc test and presented as means with 95% confidence intervals (red squares with whiskers and numbers below dot plots). Significant (*p* < 0.05) differences between groups (identified in post hoc analysis) are indicated by the same symbol type (*, ^). NRQ: normalized relative quantity.

The anatomical subsite significantly affected the fold change in *CCL2*, *CCL7*, and *CCL8* expression. The rectal polyps downregulated *CCL2* and *CCL7* by 3.1-fold and *CCL8* by 5.3-fold, while gene downregulation in the colonic polyps was absent or less substantial. Detailed analysis of the gene expression in the polyp and normal mucosa showed that the polyp expression tended to be lower in the case of the rectal location (significantly so for *CCL8*) while being relatively higher in normal mucosa (Figure 8).

A polyp location in the rectum was significantly associated with a high grade of dysplasia (*p* = 0.004), prevalence of the villous component (*p* = 0.001), and the polyp size (*p* = 0.0001). Therefore, to verify the effect of the polyp sublocation on chemokine expression and discern the independent predictors of *CCL2*, *CCL7*, and *CCL8* expression in colorectal adenomas, a multivariate analysis was conducted.

#### 3.2.8. Multivariate Analysis

A stepwise method was applied to co-analyze the effect of the dysplasia grade, adenoma growth pattern, adenoma size, number of polyps, and subsite and patient’s age and sex on *CCL2*, *CCL7*, and *CCL7* expression in colorectal adenomas.

Of those variables, *CCL2* expression was independently associated with the grade of dysplasia, growth pattern, number of adenomas, and patient’s age, which together explained 22% of the variability in gene expression. All associations were inverse; that is, *CCL2* expression in colorectal adenomas decreased along with the increasing number of polyps, and the patient’s age and was lower in adenomas with high grades of dysplasia and dominant villous components. The effect of the dysplasia grade was the strongest, and that of age was the weakest (Table 5).

Of the examined variables, *CCL7* expression in colorectal adenomas was affected solely by the growth pattern, which explained 8% of the variability in gene expression. *CCL7* decreased along with an increasing villous component (Table 5).

*CCL8* was significantly affected by the dysplasia grade and growth pattern, which explained 10% of the variability in gene expression. *CCL8* expression decreased along with an increasing villous component and was lower in adenomas with high-grade dysplasia (Table 5).

#### 3.2.9. Interrelationship between the Expression of MCP Chemokines

There was a positive correlation between *CCL2*, *CCL7*, and *CCL8* in the normal mucosa and polyps, with the correlation between *CCL2* and *CCL8* being equally strong, while those between *CCL2* and *CCL7* and between *CCL7* and *CCL8* were stronger in the polyps than the normal tissue (Table 6).

### 3.3. Interrelationship between the Expression of MCP Chemokines at the Protein and Transcriptional Levels

**Figure 9:**
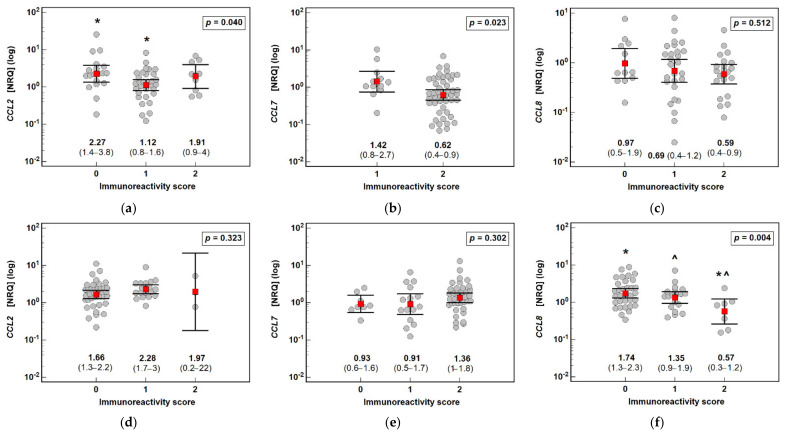
Correlation between the expression of MCP chemokines at the transcriptional and protein levels: (**a**) *CCL2* in polyps; (**b**) *CCL7* in polyps; (**c**) *CCL8* in polyps; (**d**) *CCL2* in normal mucosa; (**e**) *CCL7* in normal mucosa; and (**f**) *CCL8* in normal mucosa. Data analyzed as logarithms using one-way ANOVA with a Student–Newman–Keuls post hoc test and presented as means with 95% confidence intervals (red squares with whiskers and numbers below dot plots). Significant (*p* < 0.05) differences between groups (identified in post hoc analysis) are indicated by the same symbol type (*, ^). NRQ: normalized relative quantity.

*CCL2* and *CCL7* mRNA expression was inversely related with the CCL2 and CCL7 immunoreactivity score in the polyps, as indicated by the lower NRQ values accompanying score 1 compared with score 0 (*CCL2*) or score 2 compared with score 1 (*CCL7*). *CCL8* mRNA expression decreased along with increasing CCL8 immunoreactivity, which was non-significant in the polyps but significant in the adjacent normal tissue (Figure 9).

## 4. Discussion

There is growing interest in inflammatory mediators as potential targets for CRC chemoprevention, evoked by the efficacy of aspirin and non-aspirin NSAIDs at reducing the incidence, number, and size of neoplasm evidenced in animal models of colorectal carcinogenesis as well as human studies (reviewed in [1]). Apart from interfering with the COX/PGE2 pathway, NSAIDs are believed to act by affecting other tumor-promoting signaling pathways, altering the gut microbiome, and disrupting the inflammatory microenvironment (reviewed in [15]). Among other effects, NSAIDs can modulate the expression of MCP chemokines (manuscript submitted). The key representative, MCP-1/*CCL2*, has previously been demonstrated to be upregulated in CRC at the local [21,22] and systemic levels [30] and induce the proliferation [31] and epithelial-mesenchymal transition of tumor cells [19]. Moreover, circulating MCP-1 has been indicated as a prognosticator of increased CRC risk [32] and a component of cytokine panels differentiating CRC patients from those with high-risk conditions such as IBD and adenomas [33]. However, data on MCP-1/*CCL2* expression in colorectal adenomas are limited, not allowing confirmation of the chemokine potential as a target for chemoprevention. Only recently, comparative RNAseq analysis of the colorectal transcriptome of patient-matched (*n* = 5) samples of normal mucosa, adenomas, and adenocarcinomas has shown that *CCL2* expression is significantly upregulated in tumors compared with adenomas but downregulated in adenomas compared with normal mucosa [27]. The results presented in the current study corroborate observations regarding adenomas on a larger set of samples, indicating a similar level of downregulation. They also link a magnitude of downregulation with a polyp’s potential for malignancy. The risk of transformation into carcinoma is higher in patients with multiple polyps and in the case of larger lesions. For adenomas, this depends on the dysplasia grade as well as the contribution of the villous growth pattern. Large, predominantly villous adenomas with high-grade dysplasia are referred to as “advanced adenomas” [34]. Hyperplastic polyps, in turn, are representatives of serrated polyps [3] and have previously been considered innocuous [34]. However, recent evidence indicates that they may progress to serrated adenomas and then cancer over time [3]. Here, we showed that *CCL2* expression in polyps and the polyp-to-normal expression ratio were lower in patients with large and multiple polyps and adenomas with a dominant villous growth pattern and high-grade dysplasia. Moreover, the dysplasia grade, growth pattern, and number of polyps were, together with the patient’s age, independent predictors of *CCL2* expression. Likewise, *CCL7* and *CCL8* expression was downregulated in polyps compared with the patient-matched normal mucosa, the most pronounced example being in the case of *CCL8*, both herein and in the RNAseq analysis of Hong et al. [27]. Their expression in the polyp and expression rate mimicked the *CCL2* patterns; they were lower in the larger polyps and adenomas with a higher grade of dysplasia and decreased with the increasing contribution of the villous growth pattern. *CCL8* expression was also significantly lower in patients with multiple polyps. A dominant villous growth pattern for both *CCL7* and *CCL8* and high-grade dysplasia for *CCL8* were independent predictors of chemokine expression, although their contributions to the expression variability were rather low and indicative of the presence of other effectors which were not evaluated in the current study. The *CCL2*, *CCL7*, and *CCL8* expression seemed to depend on the polyp location, as the lowest expression ratios were observed for the rectal polyps. However, as indicated by the results of the multivariate analysis, the neoplasm location lost significance when co-examined with the histopathological findings.

The role of MCP-2/*CCL8* and MCP-3/*CCL7* in colorectal carcinogenesis and cancer progression is less studied and clear. Clinical studies have linked MCP-3/*CCL7* overexpression with liver metastasis [35], while cell culture studies have demonstrated MCP-3 to promote cancer cell proliferation in addition to enhancing their migratory and invasive properties [36]. MCP-3 also displays immunomodulatory properties and facilitates tumor growth by attracting monocytes and promoting their phenotypic transformation to tumor-associated macrophages, but it can also contribute to tumor infiltration with tumor-suppressing subsets of T lymphocyte and dendritic cells [37]. Accordingly, *CCL7* transfection has inhibited the growth of primary tumors and prevented metastasis [25,26]. In osteosarcoma [38] and melanoma [39], high *CCL8* expression has been a good prognosticator and correlated with tumor infiltration with CD8+ T cells and M1 macrophages [38], while in animal models, MCP-2/*CCL8* negatively affected the proliferation of melanoma cells and reduced the number of liver metastases [40]. On the other hand, *CCL8* has been found to facilitate the progression of glioblastoma by promoting invasion and stemness [41], pancreatic ductal adenocarcinoma by stimulating proliferation and invasiveness [42], and breast and lung cancer by recruiting Tregs into metastatic sites [16]. Corroborating its pro-tumorigenic character, overexpression of *CCL8* in breast and endometrial cancer has been predictive of poor prognoses [43]. Regarding CRC, MCP-2/*CCL8* derived from cancer-associated fibroblasts have been shown to stimulate the proliferation of cancer cells [31], while MCP-2/*CCL8* and MCP-3/*CCL7* derived from tumor-associated macrophages attract anti-tumor CD4+ and CD8+ T cells [44].

Intriguingly, all MCP proteins were upregulated in the polyps compared with the patient-matched normal mucosa, although their corresponding transcripts demonstrated unanimous downregulation. As the results of transcriptional analysis depend on genes used as normalizers, we used several internal controls, namely the most popular (*GAPDH*), the pair of genes previously found to be stably expressed in colorectal cancer (*PPIA* and *RPLP0*) [28], and *RN18S1*, which all yielded consistent results. As mentioned earlier, chemokine downregulation in the paired analysis of adenoma and normal mucosa from five patients was also noted in the study by Hong et al. [27]. Here, we demonstrated that gene expression was inversely correlated with immunoreactivity toward chemokine proteins. There are several possible explanations for the observed counter-regulation of *CCL2*, *CCL7*, and *CCL8* transcripts and MCP-1, MCP-3, and MCP-2 proteins. First, different fragments of polyps were analyzed in the transcriptomic and protein analysis, and while IHC semi-quantitatively detected the MCP content solely in the epithelial cells, the relative transcript number was determined fully quantitatively but in tissue fragments that were heterogenous and unspecified in terms of cellular composition. Secondly, there might be an expression gradient formed across the longitudinal section of the polyp resembling an expression gradient formed by MCP-2/*CCL8* in breast cancer, with an increasing chemokine concentration from the neoplastic epithelium via stroma to the periphery [45]. The prolonged half-life of MCP proteins and possible feedback inhibition is yet another plausible explanation for the observed discrepancy supported by our recent findings regarding polyp expression of heat shock proteins (HSP)-70 and 90 (manuscript submitted) and atypical chemokine receptors [7]. The HSPs play a role in carcinogenesis as chaperones of cancer-promoting proteins including MCP, protecting them from degradation and thus increasing their longevity, leading to accumulation [46,47]. Indeed, we have found *HSPA1* (HSP70) and *HSP90AA1* expression in patients with polyps to be upregulated and positively correlated with the dysplasia grade and villous component. We have also observed a downregulation of the decoy chemokine receptors *ACKR2* and *ACKR4* [7], which are involved in scavenging chemokines and directing them to degradation [48]. Lower expression of ACKRs would therefore translate into reduced chemokine turnover and further contribute to their accumulation. Like for the analyzed chemokines, ACKR downregulation has been the greatest in adenomas with the highest malignancy potential. Moreover, a recent study by Smit et al. [49] indicated that mutations in the *APC*, *TP53*, *KRAS*, and *SMAD4* genes, all characteristics for colorectal adenoma-carcinoma transition, are associated with enhanced global translational capacity, which may, at least in part, counterbalance the reduced transcription observed here.

## 5. Conclusions

Colorectal polyps are characterized by a significantly higher content of MCP-1, MCP-2, and MCP-3 proteins but a lower number of the respective *CCL2*, *CCL7*, and *CCL8* transcripts, the downregulation of which is more pronounced in polyps with greater potential for malignancy. Considering a discrepancy between mRNA and protein expression, which an enhanced translational capacity and reduced protein degradation might contribute to, chemopreventive strategies directed against chemokine proteins or molecules contributing to their accumulation, such as HSPs and ACKRs, or components of the translational machinery, rather than targeting chemokine gene expression, should be considered.

## Figures and Tables

**Table 1:** Characteristics of patients with colorectal polyps.

Parameter	IHC Cohort	RTqPCR Cohort
*n*	62	173
Sex distribution (F/M), *n*	27/35	78/95
Age (years), mean (95%*CI*)	62.9 (60.2–65.7)	65.3 (63.6–67.0)
Polyp histology, *n*		
hyperplastic polyps	4	11
tubular adenoma	23	37
tubulo-villous adenoma	29	107
villous adenoma	6	13
adenocarcinoma in the polyp	(1)	5
Grade of dysplasia, *n*		
low	55	128
high	3	29
Polyp size, *n*		
<10 mm	19	39
10–19 mm	24	75
≥20 mm	19	58
Polyp location, *n*		
right colon	12	90
left colon	35	38
rectum	15	45
Number of polyps, *n*		
single	60	129
multiple (≥2)	2	36
carpet-like lesions	0	7

*n*: number of observations; F/M: female-to-male ratio; yrs.: years; CI: confidence interval; IHC: immunohistochemistry; RTqPCR: reverse-transcribed quantitative (real-time) polymerase chain reaction (transcriptomic analysis).

**Table 2:** Association of polyp *CCL2*, *CCL7*, and *CCL8* expression with polyp characteristics.

Pathology	*CCL2* Immunoreactivity Score 0/1/2, *n*	*CCL7* Immunoreactivity Score 0/1/2, *n*	*CCL8* Immunoreactivity Score 0/1/2, *n*
Histology:	*p* = 0.956 ^1^	*p* = 0.212 ^1^	*p* = 0.600 ^1^
hyperplastic	0/4/0	0/0/4	0/3/1
tubular	8/10/5	0/7/16	4/8/11
tubulo-villous	10/15/4	0/5/24	7/14/8
villous	2/3/1	0/0/6	2/2/2
Dysplasia:	*p* = 0.033	*p* = 0.368	*p* = 0.547
low grade	20/27/8	0/12/43	13/22/20
high grade	0/1/2	0/0/3	0/2/1
Size:	*p* = 0.429	*p* = 0.260	*p* = 0.267
<10 mm	8/7/4	0/6/13	6/5/8
10–19 mm	8/12/4	0/3/21	5/13/6
≥20 mm	4/13/2	0/3/16	2/9/8
Anatomical site:	*p* = 0.769	*p* = 0.837	*p* = 0.842
right colon	3/7/2	0/3/9	3/4/5
left colon	11/17/7	0/6/29	6/16/13
rectum	6/8/1	0/3/12	4/7/4

*n*: number of observations. ^1^ Analyzed for adenomas (without hyperplastic polyps). Data analyzed using a chi-squared test.

**Table 3:** *CCL2*, *CCL7*, and *CCL8* expression in colorectal adenomas.

	Patients from IHC Cohort, *n* = 62		Patients from Whole RTqPCR Cohort, *n* = 173	
	*PPIA/RPLP0*	*GAPDH*	*PPIA/RPLP0*	*GAPDH*
*CCL2*	↓1.2, *p* = 0.209	↓1.2, *p* = 0.245	↓1.4, *p* = 0.037	↓1.5, *p* = 0.066
*CCL7*	↓1.5, *p* = 0.012	↓1.5, *p* = 0.032	↓1.7, *p* = 0.0001	↓1.9, *p* = 0.005
*CCL8*	↓2.0, *p* < 0.001	↓2.0, *p* = 0.002	↓2.3, *p* < 0.0001	↓2.5, *p* = 0.0001

Data presented as a fold change in expression in the polyps compared with the patient-matched normal mucosa, with ↓ indicating a downregulation. Data were analyzed as logarithms using a *t*-test for paired samples. IHC: immunohistochemistry; RTqPCR: reverse-transcribed quantitative (real-time) polymerase chain reaction; *n*: group size.

**Table 4:** Effect of cumulative risk of adenoma-to-adenocarcinoma transformation on expression of MCP chemokines.

Gene	FC (P/N)	Polyp (*p*)	Normal (*n*)
*CCL2*	−0.32, *p* = 0.0001	−0.46, *p* < 0.0001	ns
*CCL7*	−0.36, *p* < 0.0001	−0.32, *p* = 0.0001	0.18, *p* = 0.032
*CCL8*	−0.25, *p* = 0.003	−0.30, *p* < 0.001	ns

Data presented as Spearman correlation coefficients (*ρ*). FC: fold change; ns: non-significant (*p* > 0.05).

**Table 5:** Regression models explaining expression of MCP chemokines in colorectal adenomas.

Dependent Variable	Explanatory Variables	Regression Coefficient (*β*), *p*	*r*_p_	VIF	R^2^; ANOVA
*CCL2* (log)	(constant)	1.88			R^2^ = 0.22; *F* = 9.63, *p* < 0.0001
	dysplasia grade	−0.41, *p* = 0.003	−0.25	1.10	
	number of polyps	−0.16, *p* = 0.017	−0.20	1.02	
	growth pattern	−0.27, *p* = 0.004	−0.24	1.10	
	patient’s age	−0.01, *p* = 0.011	−0.21	1.02	
*CCL7* (log)	(constant)	0.36			R^2^ = 0.08; *F* = 12.0, *p* < 0.001
	growth pattern	−0.29, *p* < 0.001	−0.29	1	
*CCL8* (log)	(constant)	0.65			R^2^ = 0.10; *F* = 7.97, *p* < 0.001
	dysplasia grade	−0.33, *p* = 0.027	−0.19	1.10	
	growth pattern	−0.25, *p* = 0.013	−0.21	1.10	

Data were analyzed using the stepwise method of linear multivariate regression. The adenoma size (as a continuous variable), number of polyps (as a continuous variable), growth pattern (tubular adenomas coded as 1, tubulo-villous as 2, and villous as 3), grade of dysplasia (low-grade dysplasia coded as 1 and high grade as 2), and anatomical subsite (rectum coded as 1 and colon coded as 0) as well as the patient’s age and sex (male coded as 1 and female as 0) were entered into the analysis. Results are presented as regression coefficients β together with a corresponding p value, partial correlation coefficient (rp), and variance inflation factor (VIF; multicollinearity indicator) for each explanatory variable retained in the regression model and as the model’s coefficient of determination (R2) together with ANOVA results (F statistics and p value).

**Table 6:** Interrelationship between *CCL2*, *CCL7*, and *CCL8* expression.

Gene	FC (P/N)		Polyp (*p*)		Normal (*n*)	
	*CCL7*	*CCL8*	*CCL7*	*CCL8*	*CCL7*	*CCL8*
*CCL2*	0.48 ^1^	0.63 ^1^	0.52 ^1^	0.54 ^1^	0.31 ^1^	0.55 ^1^
*CCL7*		0.63 ^1^		0.66 ^1^		0.48 ^1^

Data analyzed as logarithms and presented as Pearson moment correlation coefficients (*r*). ^1^ *p* ≤ 0.0001. FC: fold change.

## Data Availability

Not applicable.
